# Reduction of Experimental Cerebral Malaria and Its Related Proinflammatory Responses by the Novel Liposome-Based *β*-Methasone Nanodrug

**DOI:** 10.1155/2014/292471

**Published:** 2014-07-14

**Authors:** Jintao Guo, Judith H. Waknine-Grinberg, Andrew J. Mitchell, Yechezkel Barenholz, Jacob Golenser

**Affiliations:** ^1^Department of Pathology and Bosch Institute, The University of Sydney, Sydney, NSW 2006, Australia; ^2^Laboratory of Membrane and Liposome Research, Department of Biochemistry, The Hebrew University-Hadassah Medical School, 91120 Jerusalem, Israel; ^3^Department of Microbiology and Molecular Genetics, The Kuvin Center for the Study of Infectious and Tropical Diseases, The Hebrew University-Hadassah Medical School, 91120 Jerusalem, Israel

## Abstract

Cerebral malaria (CM) is a severe complication of and a leading cause of death due to *Plasmodium falciparum* infection. CM is likely the result of interrelated events, including mechanical obstruction due to parasite sequestration in the microvasculature, and upregulation of Th1 immune responses. In parallel, blood-brain-barrier (BBB) breakdown and damage or death of microglia, astrocytes, and neurons occurs. We found that a novel formulation of a liposome-encapsulated glucocorticosteroid, *β*-methasone hemisuccinate (nSSL-BMS), prevents experimental cerebral malaria (ECM) in a murine model and creates a survival time-window, enabling administration of an antiplasmodial drug before severe anemia develops. nSSL-BMS treatment leads to lower levels of cerebral inflammation, expressed by altered levels of corresponding cytokines and chemokines. The results indicate the role of integrated immune responses in ECM induction and show that the new steroidal nanodrug nSSL-BMS reverses the balance between the Th1 and Th2 responses in malaria-infected mice so that the proinflammatory processes leading to ECM are prevented. Overall, because of the immunopathological nature of CM, combined immunomodulator/antiplasmodial treatment should be considered for prevention/treatment of human CM and long-term cognitive damage.

## 1. Introduction

Human cerebral malaria (CM) caused by* Plasmodium falciparum* is a major cause of malaria mortality. Survivors of CM experience developmental and behavioral impairments [1, 2]. Therefore, antiplasmodial treatment may not be sufficient for preventing cognitive CM consequences.

There is common agreement that CM is the result of cerebral vascular obstruction by parasitized erythrocytes as well as additional factors, such as immunopathological components [3, 4]. Experimental cerebral malaria (ECM) depicted in mice is a reliable model for unraveling CM pathogenesis [5] and can be prevented by immunomodulation [6–9]. In general, infection of C57Bl/6 mice with low inoculums of* P. berghei* ANKA- (PbA-) infected erythrocytes (PE) (5 × 10^4^–10^5^) leads to experimental cerebral malaria (ECM) [10]. ECM progresses rapidly, with death occurring at relatively low parasitemias approximately one week after inoculation. The mortality rate due to ECM is at least 90%; surviving mice later succumb to severe anemic malaria at high parasitemias [11].

It is well established that, in the context of neuroinflammatory diseases, glucocorticosteroids modulate T cells, macrophages, microglia, and the blood brain barrier (BBB) [12]. In a previous study, we demonstrated that administration of a novel liposomal nanodrug based on the steroid *β*-methasone hemisuccinate (BMS) as its active pharmaceutical ingredient (API) (also referred to as nanosterically stabilized liposomes (nSSL-BMS)) prevents murine cerebral symptoms and creates the therapeutic time-window necessary for administration of effective antiplasmodial drugs. Brain histology and RNA expression of cytokine-related genes suggest that nSSL-BMS alleviates the immunopathological processes that induce ECM [6]. In these experiments, murine immune status was examined at a single time point (9 days after inoculation, the peak of cerebral disease); in addition, cytokine and chemokine levels were measured only indirectly, via mRNA levels. As RNA levels do not necessarily correlate with protein levels [13], analysis of cytokine and chemokine protein levels at several time points is essential. Our current study was designed to address these limitations, thereby enabling a better understanding of the mechanism of action of the steroidal nanodrug. The results indicate the role of integrated immune responses in ECM induction and show that the new steroidal nanodrug nSSL-BMS reverses the balance between the Th1 and Th2 responses in malaria-infected mice, preventing the proinflammatory processes leading to ECM.

## 2. Materials and Methods

### 2.1. Preparation of nSSL-BMS

nSSL-BMS was prepared and characterized according to our previously published protocols [6, 14]. The nSSL-BMS were composed of HSPC/cholesterol/PEG-DSPE at a mole ratio of 55 : 40 : 5 hydrated with 250 mM calcium acetate, with a mean size of 82.2 ± 0.73 d*·*nm (polydispersity index of 0.12 ± 0.01) and a drug to lipid mole ratio of 0.17 ± 0.06.

### 2.2. Ethics Statement

All procedures adhered to the Australian National Health and Medical Research Council guidelines for animal research and were approved by both the University of Sydney Animal Ethics Committee and the Animal Ethical Care Committee of The Hebrew University of Jerusalem.

### 2.3. Animals

Six- to 8-week-old female C57BL/6 mice were obtained from the Australian Animal Research Centre (Perth, Australia) and housed in the Medical Foundation Building Animal House, University of Sydney (Sydney, Australia), in group cages under a 12 h light-dark cycle with food and water ad libitum.

### 2.4. Induction of Experimental Cerebral Malaria (ECM)

*Plasmodium berghei* ANKA (PbA) was maintained in vivo by serial transfer of parasitized erythrocytes (PE) from infected to naïve mice. Intraperitoneal injection of experimental mice with PbA from peripheral blood of infected donor mice at an inoculum of 5 × 10^4^ PE resulted in >90% incidence of terminal ECM signs on days 7-8 after inoculation (PoI), at parasitemias of up to 25% (mostly below 20%). Parasitemia was monitored by thin blood smears prepared from tail blood. These were stained with a 25% Giemsa solution, examined under a light microscope, and parasitemia determined as the percentage of infected red blood cells per 10,000 erythrocytes. ECM was determined according to neurological symptoms, that is, presentation of one or more signs of neurological deficit including ataxia, convulsions, limb paralysis, poor righting reflex, roll-over, and coma. Brains were selected at random for histology. Brain pathology observed in mice dying of ECM (generally on day 7 PoI) included hemorrhages and mononuclear cell accumulation in small vessels. In the absence of neurological symptoms, on average at 14 days PoI, nontreated mice succumbed to hyperparasitemia (above 25%) and severe anemic malaria (SM), as has been reported in other cases where mice are resistant to* P. berghei* ANKA-induced ECM [15].

### 2.5. Treatment Protocols and Mouse Euthanasia

Infected mice were administered 20 mg/kg nSSL-BMS or equal amounts of empty liposomes by i.v. injection on days 3, 5, and 7 PoI. Infected control mice were injected with 5% dextrose or equal amounts of empty liposomes. The empty liposomes were injected in a separate experiment. At least five mice from each group were chosen at random and deeply anesthetized, and brains were removed following intracardial perfusion with 20 mL cold PBS, snap frozen in liquid nitrogen, and stored at −80°C until use.

### 2.6. Quantification of Total Brain Soluble Proteins

Frozen brains were homogenized in 0.5 mL 1% (v/v) protease inhibitor cocktail solution in phosphate buffered saline (PBS) by a bullet blender, using zirconium beads. Homogenates were centrifuged at 12,000 rpm (17,200 ×*g*) for 10 minutes at 4°C. The supernatant containing soluble proteins (~200 *μ*L) was stored at −80°C until analysis.

Total soluble protein in each sample was measured using the bicinchoninic acid assay (BCA, Pierce Biotechnology, USA); 25 *μ*L sample was added to a 96-well flat bottom plate in duplicate and incubated with 200 *μ*L working reagent at 37°C for 30 min in the dark. An intense purple color developed and the absorbance was recorded by a SpectraMax 190 spectrophotometer (Molecular Devices, USA) at 562 nm. Protein concentration in unknown samples was estimated by comparison to a standard curve of bovine serum albumin (BSA).

### 2.7. Brain Cytokine Measurement

Murine brain cytokine proteins interferon gamma (IFN-*γ*), tumor necrosis factor (TNF), monocyte chemoattractant protein-1 (CCL-2/MCP-1), and monokine induced by interferon gamma (CXCL-9/MIG) were measured using the Cytometric Bead Array Flex Set (BD Biosciences). Briefly, antibody-conjugated beads (1 : 50 dilution in 5 *μ*L Capture Bead Diluent) were incubated with 5 *μ*L protein sample for one hour in a 96-well round bottom plate, at room temperature. Phycoerythrin-labelled detection fluorescent antibodies (1 : 50 dilution in 5 *μ*L Detection Reagent Diluent) were added to detect the corresponding bead-protein complexes. The plate was further incubated for one hour at room temperature in the dark and detected by a Beckman Coulter cytomics FC500 MLP flow cytometer. Data analysis was performed using FlowJo software (Tree Star, Inc). The concentration of each individual cytokine was revealed by the intensity of fluorescence of the relevant bead population compared to the standard curves.

Murine cytokine CXCL-10/IP-10 was measured by the Mouse CXCL-10/IP-10 DuoSet ELISA Development Kit (R&D Systems, USA) according to the manufacturer's protocol. Briefly, a NUNC immunoplate (96-well flat) (Thermoscientific, Australia) was coated with capture antibody in PBS overnight at room temperature. The plate was washed three times with PBS containing 0.05% (v/v) Tween 20 in a blocking buffer, 1% w/v BSA in PBS, was added and the plate incubated at room temperature for 1 h, followed by rinsing three times with washing buffer. Brain protein samples in PBS and CXCL-10/IP-10 standards were plated in duplicate and incubated for 2 h at room temperature, following which unbound proteins were removed by repetitive washes with washing buffer. Biotinylated goat anti-mouse CXCL-10/IP-10 detection antibody was added to detect the bound proteins. Samples were further incubated at room temperature for 2 h and washed three times and the protein-antibody complex was labeled with diluted Streptavidin-horseradish peroxidase (HRP, R&D Systems, USA) for 20 min in the dark at room temperature. After three washes, tetramethylbenzidine (TMB) (Sigma, USA) was added and the plate was incubated for an additional 20 min. The reaction was stopped by addition of sulfuric acid. Absorbance was read at 450 nm by a SpectraMax 190 spectrophotometer (Molecular Devices, USA). The concentration of CXCL-10/IP-10 was estimated from the standard curve using GraphPad Prism 5 software.

Cytokine concentrations estimated were normalized to the mg total protein.

### 2.8. Statistics

In vivo and cytokine results were evaluated for statistical significance using one-way ANOVA and Tukey test in GraphPad Prism and presented as mean ± S.E.M. Significant differences of *P* < 0.05 are noted by an asterisk.

## 3. Results

**Figure 1:**
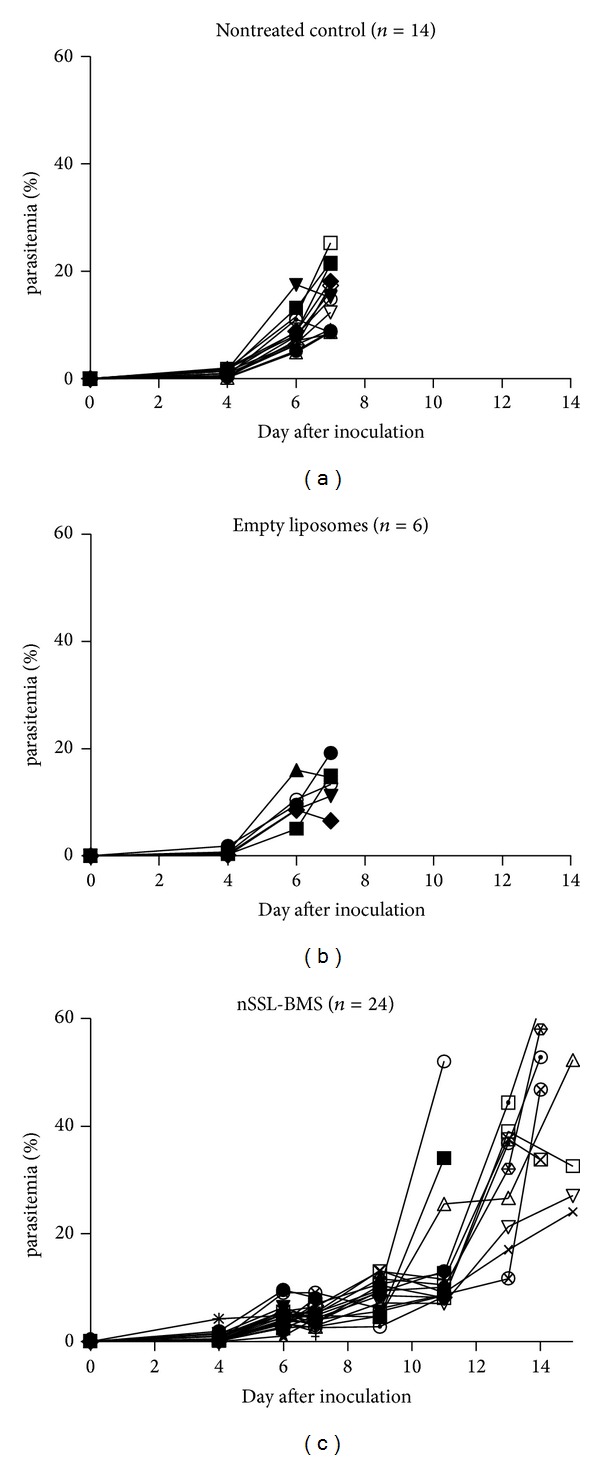
Effect of nSSL-BMS on murine PbA infection. Each line represents a single mouse. The empty liposomes were examined in a separate experiment. The results in the nSSL-BMS injected mice are significantly different compared to the controls (survival and parasitemia on days 8–14 PoI).

We found that nSSL-BMS consistently prevented ECM; nontreated mice and mice treated with empty liposomes developed irreversible cerebral disease, while nSSL-BMS treated mice did not show ECM symptoms and later developed severe life-threatening malaria (characterized by anemia) (Table 1). The control mice developed irreversible CM within a week PoI, with relatively low parasitemias (less than 25%, mostly below 20%). Mice which survived ECM following nSSL-BMS administration developed severe malaria and were euthanized a week later (two weeks PoI), at parasitemias above 35% (Figure 1).

**Figure 2:**
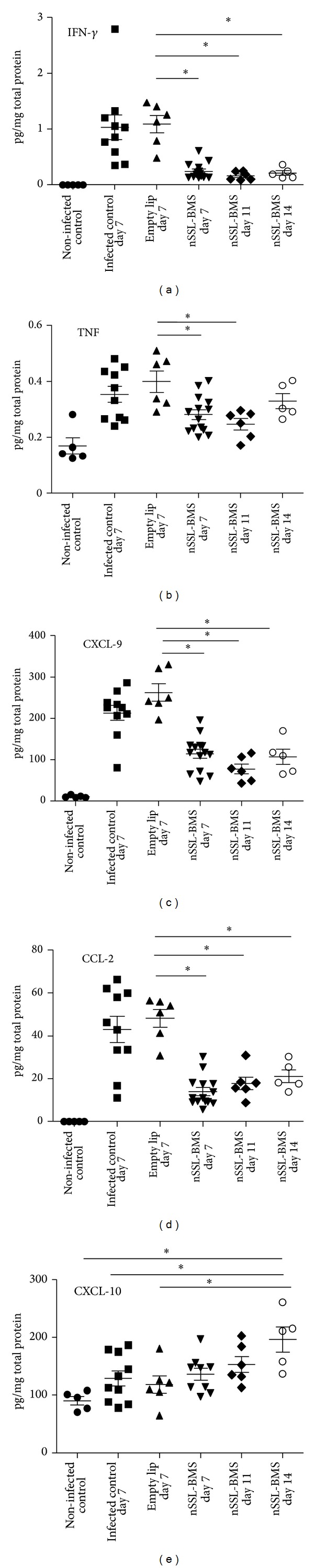
Brain cytokine and chemokine levels in PbA-infected mice treated with nSSL-BMS. Each symbol represents a single mouse. *Significant, *P* value < 0.05. Noninfected control, *n* = 5. Infected control day 7, *n* = 10. Empty nSSL day 7, *n* = 6. nSSL-BMS day 7, *n* = 9. nSSL-BMS day 11, *n* = 6. nSSL-BMS day 14, *n* = 5.

On day 7 PoI, the brains of moribund control mice contained elevated levels of all tested proinflammatory proteins (Figure 2). In contrast, the brains of nSSL-BMS-treated mice displayed much lower levels of the proinflammatory cytokines and chemokines IFN-*γ*, TNF, CCL-2, and CXCL-9. These relatively low levels remained stable throughout the experiment. CXCL-10 levels were similar in all groups of infected mice. On day 14 PoI, CXCL-10 levels in nSSL-BMS-treated mice were slightly elevated (all other mice were moribund due to ECM and were euthanized) (Figure 2).

## 4. Discussion

In the context of neuroinflammatory diseases, glucocorticosteroids (GC) modulate T cells, macrophages, microglia, and the blood brain barrier [12]. A previous study using the murine model of CM showed that, in contrast to free *β*-methasone hemisuccinate (BMS), a novel superior steroid formulation, liposomal BMS (nSSL-BMS), prevented ECM and created a therapeutic time-window that enabled antiplasmodial treatment and total cure. Brain histology and mRNA measurements of cytokine and chemokine related genes suggested that nSSL-BMS alleviates the immunopathological process that induces ECM [6]. While cytokines and chemokines may reach the brain through the damaged blood brain barrier that accompanies ECM [16], local production of cytokines and chemokines in CM has been emphasized as the important triggering element [17–20]. The examined samples were taken after thorough blood perfusion, indicating that the results most likely reflect the activity of molecules that either were produced in the brain or were produced elsewhere but were bound to local receptors. In addition, this murine model depicts brain damage without respiratory stress, a remote syndrome, thus excluding possible effects of systemic damage. Therefore, the current study stresses the role of cerebral immune responses mediated by actual Th1-related proteins in the initiation of ECM. The results demonstrate that the new steroidal nanodrug nSSL-BMS reverses the balance between the Th1 and Th2 responses so that the inflammatory process and the corresponding ECM are prevented.

It is common practice to measure gene expression levels via mRNA expression, as in our previous study using this mouse model [6]. However, RNA levels may not reflect the presence and levels of actual cytokines and chemokines due to the many processes which occur between transcription and translation. Protein stability is a significant factor; the half-life of different proteins can vary from minutes to days, whereas the degradation rate of mRNA falls within a much tighter range (2–7 hrs for mRNAs versus 48 hrs for proteins). The biochemical diversity of proteins means that individual correlation levels with associated mRNA are likely to vary widely [13]. Therefore, in order to complete the understanding of the mechanism of action of our steroidal nanodrug, in this study, we measured actual levels of the relevant cytokines and chemokines.

We selected IFN*γ* and TNF (proinflammatory cytokines) and CCL-2, CXCL-9, and CXCL-10 (proinflammatory chemokines) as representative molecules for examining the relationship between the level of Th1-related immune responses and ECM occurrence in mice treated by nSSL-BMS. IFN*γ* and TNF are central factors in the cascade of events leading to human CM [21, 22] and ECM [23–25]. IFN*γ* induces TNF production, which in turn upregulates adhesion molecules on brain endothelial cells, leading to an increase in the adhesion of platelets and parasitized erythrocytes [26]. CCL-2, which recruits monocytes, memory T cells, and dendritic cells to inflammation sites produced by either tissue injury or infection, is implicated in the pathogenesis of several diseases characterized by monocytic infiltrates, including neuroinflammatory processes [27]. An increase in proinflammatory Th1-related chemokines, for example, CCL-2, CXCL-9, and CXCL-10, is associated with ECM [17, 28–30]. CXCL-9, related to CXCL-10, is chemoattractant for T cells and NK cells and affects the growth, movement, and activation state of cells that participate in immune and inflammatory responses [31]. In response to IFN*γ*, CXCL10 is secreted by several cell types, including monocytes, endothelial cells, and fibroblasts. CXCL10 has been associated with several roles, including chemoattraction of monocytes/macrophages, T cells, NK cells, and dendritic cells; promotion of T cell adhesion to endothelial cells; antitumor activity; and inhibition of bone marrow colony formation and angiogenesis. Several of these activities are related to many Th1-type inflammatory diseases [32]. Elevated plasma levels of CXCL10 have been tightly associated with CM mortality [33, 34].

De Miranda et al. [35] found that on day 5 PoI PbA-infected mice presented anxiety signs, histopathological alterations in the brainstem, cerebrum, and hippocampus, and increased cerebral levels of proinflammatory cytokines. These findings suggest involvement of central nervous system inflammatory mediators in the anxiety symptoms observed in CM. We found that PbA-infected nontreated mice and infected mice injected with empty liposomes presented increased levels of cerebral proinflammatory cytokines and chemokines. This was most significant on day 7 PoI, when control mice develop irreversible CM. In contrast, there was a reduction in IFN-*γ*, TNF, CCL-2, and CXCL-9 levels in mice treated with nSSL-BMS. Although CXCL-10 levels were similar in all infected groups up to day 11 PoI, an increase was observed in nSSL-BMS-treated mice (the only surviving group) on day 14 PoI. All other immune parameters in the surviving mice were relatively low and similar to previous days. Wilson et al. [33] and Campanella et al. [28] have suggested a central role for CXCL-10 in the development of ECM. Wilson et al. demonstrated decreased levels of CXCL-10 and improved survival in mice 11 days PoI, following injection of artemether and atorvastatin. In treated mice, no difference in CXCL-10 levels was noted on day 5 PoI, compared to control levels. Although these investigators relate the improved survival to reduced deleterious effects of CXCL-10, it should be stressed that the most striking event in CM induction is the impairment of the BBB, which starts early in plasmodial infection. Campanella et al. showed an increase in CXCL-10 levels at a similar, relatively late stage after infection. Moreover, their CXCL-10 deficient mice were only partially protected against CM [28]. In our experiments, treatment with nSSL-BMS did not alter CXCL-10 level until day 11 PoI; increased levels (compared to nontreated infected mice) were observed on day 14 PoI. These later days PoI are much less relevant to the induction and development of the cerebral syndrome (which was fatal in nontreated mice 6 or 7 days PoI). These results indicate that either CXCL-10 is not necessarily a major player in ECM induction or its role is masked by the activity of other immune factors.

In our experiments, a correlation was observed between the immunological effects of nSSL-BMS and ECM prevention. Although individual cytokines and chemokines may contribute to the induction of (E)CM [36, 37], the end result of malarial infection is determined by a repertoire of immune responses. A synergistic effect may increase disease severity; for example, IFN*γ* and TNF, together with other cytokines and chemokines, synergize in the upregulation of adherence molecules, which stimulate immunopathology [25, 38]. This concept applies to a variety of chemokines [28]. The opposite process—CM avoidance or alleviation—is also dependent on a battery of cytokine responses (and/or chemokines or antioxidants) rather than on a single component [2, 28, 39, 40]. In a similar fashion, the end result of immune (and antiparasitic) intervention is also a balance of synergistic and antagonistic reactions.

The relevance of CM research using mouse models has been discussed [5]. It has been emphasized that drug validation is impossible without in vivo experiments. Moreover, some newly approved therapies are based on drugs previously tested for efficacy in mice (e.g., artemisinin combination therapy). Although the efficacy of glucocorticosteroids for CM treatment has been questioned, the nanosteroidal drug nSSL-BMS is a superior, novel nanodrug which displays a lack of toxicity as well as improved delivery to the inflamed brain [6]. This compound reduced ECM and the corresponding Th1 responses, confirming the hypothesis that the cerebral syndrome is a result of a Th1 shift in the balance of Th1/Th2 responses. Adjunctive therapy coupled to antiplasmodial treatment has been shown to prevent ECM cognitive defects [2]. Because of the immunopathological nature of CM, combined nSSL-BMS/anti-plasmodial-based therapy is suggested for prevention/treatment of CM and long-term cognitive damage.

## 5. Conclusions

The results confirm the hypothesis that the cerebral syndrome in ECM is a result of a dynamic shift in the balance of Th1/Th2 responses. A novel liposome-based nanosteroidal drug, *β*-methasone hemisuccinate (nSSL-BMS), reduces both cytokine and chemokine proinflammatory responses that lead to CM in a murine model. Our steroidal nanodrug prevents ECM by inducing multiple changes in the immunopathological process; therefore, combination therapy using antiplasmodial treatment and this novel drug would be of great importance in a clinical setting.

## Figures and Tables

**Table 1:** ECM rates after treatment of PbA-infected mice with nSSL-BMS.

Group∗	^ a^ECM	SM	Survival on day 8 after inoculation
Control	38^b^ (95%)	2 (5%)	2/40 (5%)
Empty liposomes	12 (100%)	0 (0%)	0/12 (0%)
nSSL-BMS	2 (6.5%)	29 (93.5%)	29/31 (93.5%)

All results depict significant differences relating to experimental cerebral malaria (ECM) versus severe anemic malaria (SM) and nSSL-BMS versus control groups.

∗Cumulative results of three experiments.

^a^Mice were euthanized when signs indicated the onset of irreversible disease.

^b^Number of mice in the group.
